# Validation of the Padova Prognostic Score for Colitis in Predicting Long-Term Outcome After Restorative Proctocolectomy

**DOI:** 10.3389/fsurg.2022.911044

**Published:** 2022-07-25

**Authors:** Imerio Angriman, Annaclaudia Colangelo, Claudia Mescoli, Matteo Fassan, Renata D’Incà, Edoardo Savarino, Salvatore Pucciarelli, Romeo Bardini, Cesare Ruffolo, Marco Scarpa

**Affiliations:** ^1^ Clinica Chirurgica I, University Hospital of Padova, Padova, Italy; ^2^ Department of Medicine, (Pathology Section), University Hospital of Padova, Padova, Italy; ^3^ Department of Surgical & Gastroenterological Sciences, (Gastroenterology Section), University Hospital of Padova, Padova, Italy; ^4^ General Surgery Unit, Padova University Hospital, Italy

**Keywords:** ulcertive colitis, crohn's colitis, restorative proctocolectomy, padova prognostic score for colitis, pouch failure

## Abstract

**Background:**

In 10%–20% of cases it is impossible to make a differential diagnosis between ulcerative colitis and Crohn's colitis. A 50% failure rate of J pouch ilea-anal anastomosis is observed in Crohn's colitis. In 2009, we created the Padua Prognostic Score for Colitis (PPSC) to predict the long-term clinical and functional outcome and quality of life of patients undergoing restorative proctocolectomy with J pouch. The aim of the present study is to establish and validate the accuracy of a prognostic score for chronic inflammatory bowel diseases (IBD).

**Patient population and methods:**

The PPSC was created in 2009 by integrating clinical and histological information of patients undergoing RPC. It included preoperative perianal abscess or fistula, rectal sparing, terminal ileum involvement, skip lesions and histological diagnosis of indeterminate colitis or Crohn's colitis on the operative specimen. The validity of this score was tested in predicting postoperative abscess or fistula, anal canal disease, pouchitis, pouch failure and new diagnosis of Crohn's disease. Correlation analysis, ROC curve analysis and survival analysis were used to validate the PPSC in a different cohort from the previous one.

**Results:**

We retrospectively enrolled in this study 138 consecutive patients undergoing CPR for ulcerative colitis (*n* = 127) or indeterminate colitis (*n* = 11) in our institution since 2005 to 2020. In this period, we observed 11 patients with postoperative abscess or fistula, 3 with anal canal disease, 40 with pouchitis, 6 with pouch failure and 6 with new diagnosis of Crohn's disease. In the new validation cohort, the PPSC confirmed to have a good accuracy in predicting the onset of postoperative CD (AUC = 74.5%, *p* = 0.018). Kaplan Meier curves demonstrate how a PPSC over 1 can reliably predicts the long-term onset of, pouchitis (*p* = 0.002) and anal abscess or fistulae (*p* = 0.04).

**Conclusions:**

In this validation study we confirmed the accuracy of the PPSC in predicting postoperative fistulas or abscesses and pouchitis. Therefore, we believe that in clinical practice patients with a PPSC score greater than 1 should be warned of this risk of possible Crohn’s disease diagnosis and pouch failure.

## Background

Chronic inflammatory bowel diseases (IBDs) are relapsing, and remitting diseases characterized by chronic inflammation of the gastrointestinal tract and cause diarrhea and abdominal pain (1). IBDs diagnosis is based on clinical, histological, endoscopic and imaging data (2). Within the colon, they include Crohn's colitis (CC), ulcerative colitis (UC) and indeterminate colitis (IC). IC diagnosis occurs in 10%–20% of cases when it is not possible to distinguish between UC and CC (3–5) and it is more frequent in acute onset of fulminant colitis, when histological aspects of CC and UC overlap due to transmural inflammation, even in absence of granulomas. UC generally continuously affects the colon from rectum, involves only the mucosal layer of the colonic wall and can extend proximally and continuously throughout the colon mucosa (6). By contrast, CC is characterized by transmural inflammation evolving in fibrosis, stenosis and micro-perforations or fistulae, can involve the colon in a discontinuous way and can be associated to lesions within the entire digestive tract (1).

A major problem in IBD surgery concerns the right choice of procedure to perform. Restorative proctocolectomy (RPC) with J pouch is the gold standard procedure in case of UC as it significantly reduces the risk of cancer associated with UC and preserves the natural route of defecation (7–10). Otherwise, for CC this procedure usually fails with poor quality of life of the patient in 50% of cases, as it is not functional (11–15) with more than half of subjects who may finally require the removal or diversion of the pouch. In case of IC, the lack of a clear classification affects the ability to predict a long-term outcome of RPC. In IC patients, RPC seems to have a functional outcome like UC patients, although some studies have associated the IC with a higher rate of perineal complications and loss of the pouch (16–22). Although there is not much information regarding post-RPC quality of life in IC patients which is reportedly slightly worse than in UC patients (16), RPC has become the gold standard for IC (23).

In case of urgent/emergent surgery (i.e., in case of colonic perforation, massive gastrointestinal bleeding with hemodynamic instability, not responsive to treatment or toxic megacolon), a correct preoperative diagnosis is even more complex, but often also histological due to transmural inflammation, for which the diagnosis of IC is made. Several candidate biomarkers have been tested for the differential diagnosis but currently, the results are still unconclusive (24–26). For all these reasons and for the better identification of patients who would benefit in the long term from this type of procedure, we developed a prognostic score, the Padova Prognostic Score for Colitis (PPSC) in 2009 (27). This score included both preoperative clinical characteristics and histology after colectomy, to predict the long-term outcome and quality of life after RPC for UC, CC and IC. The objective of this study was to validate the effectiveness of PPSC in predicting the long-term outcome and quality of life of patients after RPC in a cohort of patients different from exploratory cohort.

## Methods

### Study Design

**Figure 1:**
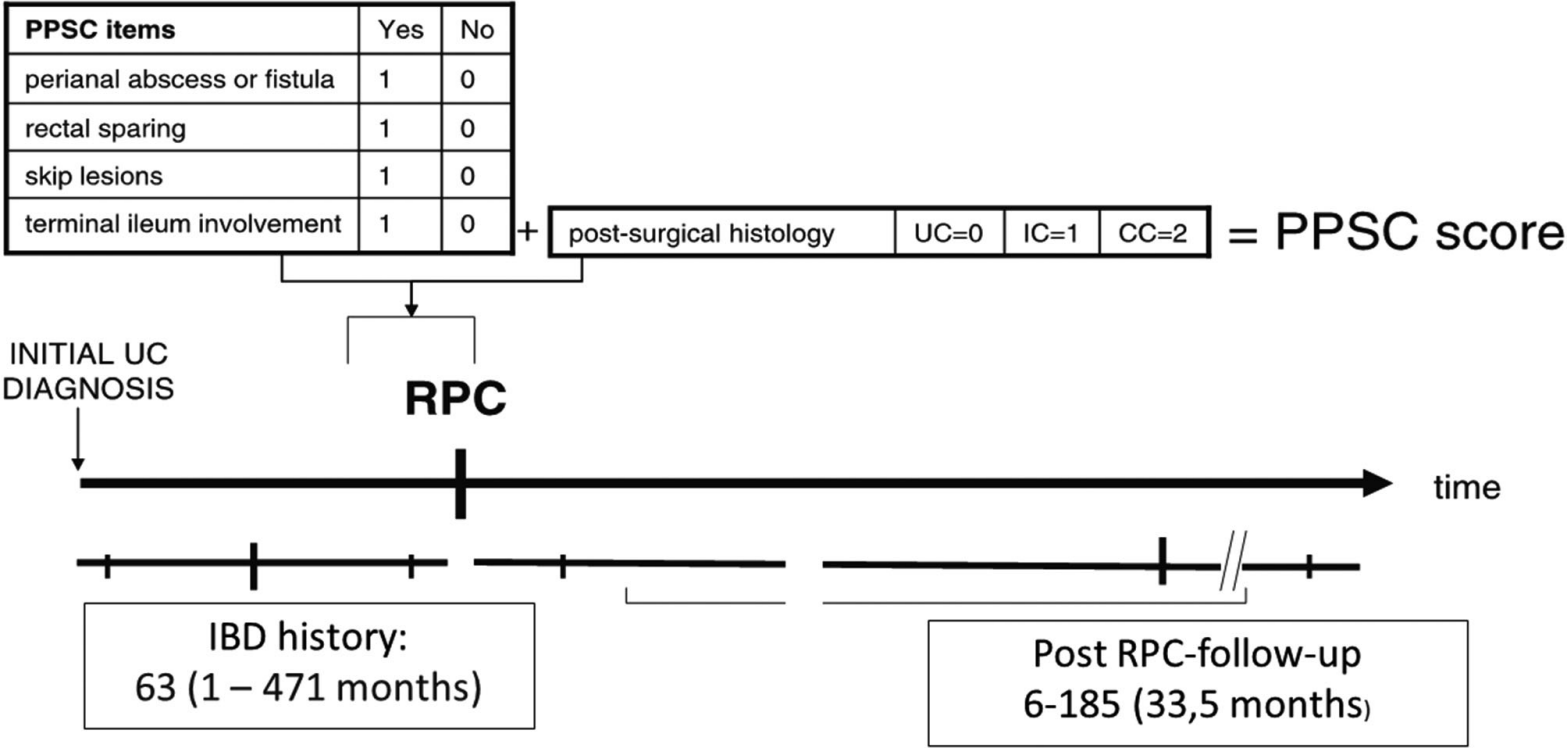
Study design.

This is a single-center, retrospective validation study analyzing the consecutive cohort of patients undergoing RPC for UC and IC at the General Surgery Unit of the Azienda Ospedale Università di Padova. Written informed consent for the use of anonymized data for scientific purposes was obtained from all patients preoperatively and the study was notified to the Ethical Committee of the Azienda Ospedale Università di Padova. Demographic and anamnestic data, and data pertaining to the surgery procedures and post-operative histological results were retrieved as well as the postoperative early and long term follow up. Patients were then followed up for at least 12 months after surgery considering the following indicators: anal fistulas, abscesses, anal fissures, pouchitis and its treatment, anal canal disease and CD diagnosis after RPC. Finally, the follow-up was assessed. Study design is shown in Figure 1.

### Patient Selection

We included in this validation study 138 consecutive patients who underwent RPC for UC or IC between 2005 and 2020. This patient cohort is temporally subsequent to the exploratory cohort used to create the original PPSC (27). All patients had a preoperative diagnosis of UC based on clinical, laboratory, imaging, and endoscopic histological data (2). All surgical procedures have been performed by an experienced surgeon in IBD surgery (IA). At the preoperative evaluation, all patients with a PPSC score greater than 1 (with at least one of the following risk factors: history of perianal abscess or fistula, rectal sparing, skip lesions, backwash ileitis or preoperative histological diagnosis of CC or IC) were accurately warned against the risk of possible postoperative Crohn’s disease diagnosis and pouch failure. Nevertheless, none of them refused to undergo restorative proctocolectomy.

### Restorative Proctocolectomy

Surgical indications and interventions are different in case of emergency, urgency or elective situations. The RPC were performed with laparoscopic or laparotomic techniques, with mechanical anastomosis and in 2 or 3 stages depending on the patient's preoperative clinical condition. Emergency surgery is required in case colonic perforation, massive gastrointestinal hemorrhage with hemodynamic instability or toxic megacolon that does not respond quickly to medical therapy. It is also necessary in case of fulminant colitis non-respondent to medical treatment. In these cases, the choice intervention is a subtotal colectomy with closure of the rectal stump and a terminal ileostomy with or without rectal stump sinking (Hartmann's pouch) or mucous fistula. This type of intervention is preferable in acute situations to improve patients’ conditions to wean drugs effects (28). The reconstructive surgery will be performed after the improvement of patient's clinical condition (29). Elective surgery should be performed for patients with persistent symptoms despite the therapy, patients with higher risk of cancer development or with dysplastic lesions (30). In this case, the main surgical procedure is RPC in 2 stages and this is the gold standard surgical procedure for UC and IC. This procedure was performed with a lateral protective ileostomy closed generally 8–12 weeks later (31). We used a J-pouch reservoir of about 15 cm and mechanical anastomosis, both with laparotomic and laparoscopic techniques.

### the Padova Prognostic Score for Colitis

PPSC is a simple clinical score that includes pre-surgical clinical variables and post-surgical histological ones (27). The first ones are history of perianal abscess or fistula, rectal sparing, skip lesions and backwash ileitis and these items could be suggestive of CC and a point is attributed for each of them if present obtaining the simple clinical PPSC. The postoperative histological variables allowed to classify the colitis in UC, CC and IC (Figure 1). The sum of clinical PPSC and histological scores give the full PPSC. The higher is the result, the worse the expected postoperative outcome will be (27). Moreover, the accuracy of the PPSC in predicting long-term outcome was assessed based on the functionality of the pouch, late complications and quality of life.

### Statistical Analysis

Data was analyzed by Microsoft Excel and Jamovi software (www.jamovi.org). Continuous data are presented as medians (IQR) and data were compared to the Mann-Whitney U-rank test, while dichotomous data are presented as frequency and Fisher's exact test was used to compare these variables. Correlations between variables were explored with the Spearman correlation rank test. Post hoc correlation power calculation and linear multiple regression analysis were performed. Complication-free survival and pouch survival were calculated using the Kaplan-Meier method with the follow-up time starting at RPC and ending at the onset of complication, first re-operation, or at the longest follow-up. The cumulative complication rates according to the various parameters were compared using the log rank test. ROCs curve analysis was assessed as described by Henderson (32) and they were used to assess PPSC accuracy in predicting postoperative diagnosis of CD and of pouchitis. Set a statistical significance level (*α*) to 0.05, a power (1 - *β*) to 0.80, an area under the curve to 0.75 and a null hypothesis value to 0.50; the resulting minimum sample size required for ROC analysis was 41 patients. A *p* level <0.05 was considered significant for all analyses.

## Results

### Patients’ Characteristics

**Figure 2:**
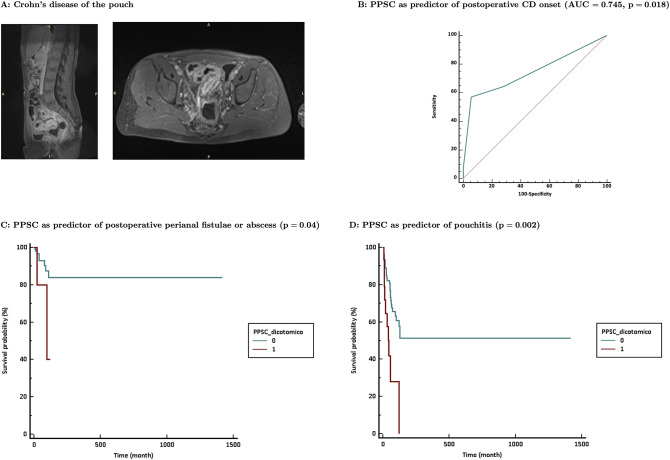
(**A**) example of Crohn’s diease localization at the ileal pouch; (**B**) PPSC as predictor of postoperative diagnosis of CD; (**C**) PPSC over 1 as predictor of anal abscess or fistulae pouchitis (*p* = 0.002) as shown in (**D**).

We retrospectively enrolled in this study 138 consecutive patients undergoing RPC for ulcerative colitis (*n* = 127) or indeterminate colitis (*n* = 11) in our institution since 2005 to 2020. They were 92 males and 46 females, with an age at diagnosis ranging from 3 to 75 years (median 36 years). The surgical indications for RPC were severe UC in 17 patients, chronic UC/IC resistant to medical therapy in 91 patients, dysplasia, or cancer in UC (21 patients) or fulminant UC (9 patients). Patients’ characteristics and surgical details and postoperative histology are shown in Table 1 and in Table 2, respectively. In the postoperative follow up, we observed 11 patients with abscess or fistula, 3 with anal canal disease, 40 with pouchitis, 6 with new diagnosis of Crohn's disease in the pouch (Figure 2A) and 6 with pouch failure. Follow up details are shown in Table 3.

**Table 1:** Patients characteristics.

Number of patients	138
Male/Female	92/46
Age at diagnosis (years)	3–75 (36)
Age at surgery (years)	15–79 (46)
Time between diagnosis and surgery (months)	1–471 (63)
Indication for intervention (patients):	
Severe UC	17
Chronic resistant UC	91
Dysplasia or cancer	21
Fulminant UC	7
Clinical diagnosis (patients):	
UC	127
IC	11
Preoperative clinical / endoscopic manifestations	
Perianal abscesses	7
Anal fistulas	4
Hemorrhoids	6
Fissures	1
Backwash ileitis and ileal inflammation over 3 cm	2
Discontinuous inflammation (skip lesions)	4
Rectal sparing	4
Preoperative medical therapy (more than one per patients is possible)	
None	2
Topical	10
Steroids	13
Azathioprine	53
Salazopyrine	2
Cyclosporine	8
Infliximab	73
Retuximab	1
Adalimumab	21
Golimumab	5
Edolizumab	2
Apheresis	1
Duration of therapy (months)	6 days–187 months
Preoperative symptoms	
Pain at diagnosis	45
No. stool / day	1–40 (9)
>3 stool / day	94
Rectorrhagia	69
Fever	22
Weight loss	32
Perianal pain	8
Extraintestinal manifestations:	
Joint manifestations (arthritis / ankylosing spondylitis)	12 (7/5)
Primary sclerosing cholangitis	4
Cutaneous manifestations (Pyoderma gangrenous/erythema nodosum)	4 (1/3)
Eyes involvement (uveitis/episcleritis)	1 (1/0)

*Data are shown as median (IQR) or frequency, as appropriate.*

**Table 2:** Surgical details and postoperative histology.

**a. Restorative proctocolectmy details**	
Type of surgery	
Two stage	109
Three stage	29
First stage open / laparoscopic	85/50
Interval between second and third time (months)	7 (3–79)
Second stage open / laparoscopic	24/5
Ostomy closure interval (months)	5 (1–81)
Ostomy closure study:	
Pouch endoscopy	32
Barium enema	122
**b. Histology on the colectomy specimen**	
TNM staging (if cancer)	
Dysplasia / microadenoma	12
NET / muICnous appendage	3
T1N0	5
T2N2b	2
T3N0-N2b	1-Mar
T4N2b	1
Neoadjuvant / adjuvant therapy (se k)	7-Jan
Histological diagnosis:	
UC	108
IC	21
CD	9 (2 UC-like)
Histological features:	
Transmural phlogosis	56
Discontinuous inflammation	5
Granulomas	6
Ileal inflammation	32
**c. Early post operative outcome**	
Postoperative complications:	
Bleeding	2
Pelvic sepsis	1
Small bowel obstruction	6
Perforation	3
Anastomotic leak	4
Other (fever / etc) ù	8
DVT / EP	2
Treatment of complications:	
Medical	11
Radiological drainage	1
Endoscopic	0
Reoperation	10

*Data are shown as median (IQR) or frequency, as appropriate.*

**Table 3:** Follow up.

follow-up (months)	33.5; IQR 6–185
**a. Functional outcome**	
No. of daily stool	2–20 (6)
Intestinal symptoms	24
Tenesmus	3
Bleeding	2
Abdominal pain	8
Diarrhea	8
Anal burning	8
Systemic symptoms	1
Continence	3
Soiling	3
Urgency	6
**b. Long term complications**	
Fistulas and anal abscesses	11
Anal fissures	5
Pouchitis and its treatment (40 pcs).	40
Anal canal disease (3 pcs)	3
CD diagnosis (6 pcs with 2 RCU-like)	6
Reoperation	37
Indications for surgery	
Incisional hernia	10
Cancer	5
Pouch failure	6
Dilations due to stenosis	7
Perineal pathology	8
Other	9

### PPSC as Predictor of Crohn's Colitis

In the new validation cohort, high PPSC score were significantly associated to postoperative development of perineal pain (R = 0.24; *p* = 0.014) and anal disease (R = 0.24; *p* = 0.003). The PPSC predicted a postoperative diagnosis of CD with a sensitivity of 57.14% (95% CI, 28.9–82.2) and a specificity of 94.35% (95% CI, 88.7–97.7); the area under the curve (C-index) was 0.745 (95% CI, 0.664–0.815; *p* = 0.0018) and the positive likelihood ratio was 10.12 (Figure 2B). At survival analysis, high PPSC scores were significantly associated to the onset of fistulas/abscesses and Kaplan-Meier curves demonstrate how a PPSC over 1 can reliably predicts the long-term risk of anal abscess or fistulae (*p* = 0.04), as shown in Figure 2C. On the other hand, it did not show any significant association with any other single symptoms (i.e., arthritis or other extra intestinal manifestations) or pouch failure (*p* = 0.13).

### PPSC as Predictor of Pouchitis

PPSC correlated significantly with pouchitis (R = 0.19; *p* = 0.02). PPSC predicted pouchitis with a sensitivity of 25% (95% CI, 12.7–41.2) and a specificity of 94.9% (95% CI, 88.5–98.3); the area under the curve was 0.599 (95% CI, 0.512–0.681; *p* = 0.07) and the positive likelihood ratio was 4.9 (Figure 12C). High PPSC score was associated to the occurrence of episodes of pouchitis (HR = 0.238, *p* = 0.0002). At survival analysis, Kaplan Meier curves demonstrate how a PPSC over 1 can reliably predicts the long-term risk of pouchitis (*p* = 0.002) as shown in Figure 2D.

## Discussion

One of the main questions in IBD surgery is the choice of the correct procedure to perform in case of colitis. RPC with J pouch is the gold standard procedure in case of UC and IC as it significantly reduces the risk of cancer associated with UC and preserves the natural route of defecation (7–10, 23). On the other hand, in case of CC there is a high risk of pouch with more than half of subjects who may finally require the removal or diversion of the pouch (11–15). On the contrary, in case of IC, RPC seems to have a functional outcome like UC patients, although some studies have associated the IC with a higher rate of perineal complications and loss of the pouch (16–22). The problem is that in a 15%–20% in case of IBD colitis the differential diagnosis is imprecise or impossible. Several candidate biomarkers have been tested for the differential diagnosis but currently, the results are still inconclusive (24–26). For all these reasons, in 2009, we had developed the PPSC score aimed to predict the long-term outcome and quality of life of patients after RPC (27), and with the present study we aim to validate this simple clinical and histological tool.

We enrolled in this study all the consecutive 138 patients that were operated on in our department after the first study (27) to have a completely different cohort of patients with a similar setting. All these patients were accurately warned against the risk of possible postoperative Crohn’s disease diagnosis and pouch failure if they presented at least one of the PPSC risk factors but, none of them refused to undergo restorative proctocolectomy. In this new series, the PPSC predicted CC diagnosis after RPC with a good accuracy while clinical PPSC had a lower accuracy. Moreover, PPSC correlates significantly with perineal pain and anal disease that, in some cases, might be manifestation of perianal CD. These results suggest that the histology is still a key point of the correct diagnosis in IBD colitis, and that the histological analysis should be performed in the whole colorectal tract (33). In fact, the diagnosis implies the detection finding in CD is epithelioid cell granuloma, a discrete collection of at least five epithelioid cells, with or without multinucleated giant cells, not related to crypt injury, and often poorly delimited, within the diseased mucosa (33). However, due to the irregular pattern of disease distribution of CC they might not be present in random biopsies obtained at colonoscopy and thus a misdiagnosis of UC or IC may occur (34).

Moreover, in our series, at survival analysis, high PPSC scores were significantly associated to the onset of fistulas/abscesses. According to Reza et al. (35), pouch anal fistulas should be classified into four distinct groups according to their aetiology: group 1, anastomotic related; group 2, inflammatory bowel disease related, with sub-classifications Crohn's (type A) and non-Crohn's (type B) in origin; group 3, cryptoglandular related; and group 4, malignancy related. A recent metanalysis on long-term outcomes following restorative proctocolectomy in adult Crohn's disease populations the rate of pelvic sepsis (fistula or abscess) was 13% (36). The PPSC score was confirmed to accurately predict Crohn's disease-related pouch anal fistula/abscess and could be used to inform patients about this specific risk. On the other hand, it was not significantly associated with pouch take down and this could be explained with the fact that not all the pouch created in CC are destined to fail (11) and some of the pouch failures were not due to CC onset but to cancer onset.

Interestingly, in our series, PPSC correlated significantly with pouchitis onset. This was rather unexpected, since a great part of the pouchitis manifestations are related to a mucosal microbiota disequilibrium and are not particularly related to a Crohn's like manifestations (37–39). In fact, the correlation coefficient between PPSC and pouchitis, and the accuracy of PPSC as predictor of pouchitis were rather low. A possible explanation of this apparent correlation might be the difficult differential diagnosis, at least at very early stages, between chronic pouchitis and Crohn's disease of the pouch and the terminal ileum. In fact, quite often it can be difficult to endoscopically explore the terminal ileum above the J pouch and if there is no suspect some Crohn's disease of the J pouch might be misdiagnosed as chronic pouchitis. On the other hand, in a recent French study, significant overlap in term of risk factors for chronic pouchitis and Crohn's disease of the ileal pouch had been observed (40). In fact, although articular manifestations and BMI were exclusively associated with Crohn's disease of the ileal pouch. Other extra intestinal manifestations and a previous diagnosis of indeterminate colitis resulted to be independent predictors both for Crohn's disease and chronic pouchitis. The authors then concluded that, taken together, these results suggest that Crohn's disease and chronic pouchitis are two entities among a continuum of chronic inflammation of the pouch (40). In our opinion, we agree that while acute pouchitis is clearly related to microbiota disequilibrium, chronic pouchitis might add an immunological unbalance but all these manifestations are mostly confined to the ileal pouch mucosa (37–39). Crohn's disease is a full thickness disease of the ileal wall and probably it can more heavily involve the immune microenvironment of every layer of the bowel wall.

The main limits of the study are its retrospective design, that is in part mitigated using a prospectively maintained database but that prevent the analysis of the PPSC as predictor of postoperative quality of life and the relatively paucity of the patients who had a postoperative diagnosis of CD that prevented a reliable and meaningful analysis on what happen to patients who had RPC in CD. The potential selection bias in patients with PPSC risk factors was avoided by the fact that all the patients, even if accurately informed on the risk of following Crohn's disease diagnosis and/or pouch failure, still preferred to undergo to restorative proctocolectomy instead of the simple proctocolectomy and end ileostomy. A second limit of the study was the lack of the analysis of the possible prediction of postoperative quality of life. Unfortunately, we did not collect quality of life data along this period. Moreover, a cross sectional analysis made in 2020–2021, when the study was performed, would yield very heterogeneous data since we included patients operated on since 2005.

In conclusion, in this validation study we confirmed the accuracy of the PPSC in predicting postoperative fistulas or abscesses, pouchitis and a new diagnosis of Crohn's disease. Therefore, we believe that in clinical practice patients with a PPSC score greater than 1 should be warned of this risk of possible Crohn’s disease diagnosis and pouch failure. PPSC may be inserted in the decision-making process when proposing a proctocolectomy to an IBD patient.

## Data Availability

The raw data supporting the conclusions of this article will be made available by the authors, without undue reservation.
